# Passive Immunization with Recombinant Antibody VLRB-PirA^vp^/PirB^vp^—Enriched Feeds against *Vibrio parahaemolyticus* Infection in *Litopenaeus vannamei* Shrimp

**DOI:** 10.3390/vaccines9010055

**Published:** 2021-01-16

**Authors:** Jassy Mary S. Lazarte, Young Rim Kim, Jung Seok Lee, Jin Hong Chun, Si Won Kim, Jae Wook Jung, Jaesung Kim, Pattanapon Kayansamruaj, Kim D. Thompson, Hyeongsu Kim, Tae Sung Jung

**Affiliations:** 1 Laboratory of Aquatic Animal Diseases, Research Institute of Natural Science, College of Veterinary Medicine, Gyeongsang National University, 501-201, 501, Jinju-daero, Jinju-si, Gyeongsangnam-do 52828, Korea; jassylazarte@yahoo.com (J.M.S.L.); yl0808@nate.com (Y.R.K.); leejs058@gmail.com (J.S.L.); hilanamang@naver.com (J.H.C.); ksw0017@hanmail.net (S.W.K.); wjdwodnr0605@gmail.com (J.W.J.); afteru70@gmail.com (J.K.); 2 Center of Excellence in Aquatic Animal Health Management, Faculty of Fisheries, Kasetsart University, 50 Ngamwongwan Rd, Ladyao, Chatuchak, Bangkok 10900, Thailand; pattanapon.k@ku.th; 3 Moredun Research Institute, Pentlands Science Park, Bush Loan, Penicuik, Midlothian EH26 0PZ, UK; Kim.Thompson@moredun.ac.uk; 4 Inland Aquaculture Research, National Institute of Fisheries Science, #55, 25gil, Yeomyeong-ro, Jinhae-gu, Changwon-si, Kyeongsangnam-do 51688, Korea; kimk2k@korea.kr; 5 Centre for Marine Bioproducts Development, Flinders University, Bedford Park, SA 5042, Australia

**Keywords:** AHPND, variable lymphocyte receptor (VLR), VLRB antibody, passive vaccine, *Vibrio parahaemolyticus*, *Photorhabdus* insect-related PirAB^vp^

## Abstract

The causative agent of acute hepatopancreatic necrosis disease (AHPND) is the bacterium, *Vibrio parahaemolyticus*, which secretes toxins into the gastrointestinal tract of its host. *Vibrio parahaemolyticus* toxins A and B (PirA^vp^/PirB^vp^) have been implicated in the pathogenesis of this disease, and are, therefore, the focus of studies developing treatments for AHPND. We previously produced recombinant antibodies based on the hagfish variable lymphocyte receptor B (VLRB) capable of neutralizing some viruses, suggesting that this type of antibody may have a potential application for treatment of AHPND. Here, recombinant PirA^vp^/PirB^vp^, produced using a bacterial expression system, were used as antigens to screen a hagfish VLRB cDNA library to obtain PirA^vp^/PirB^vp^-specific antibodies. A cell line secreting these antibodies was established by screening and cloning the DNA extracted from hagfish B cells. Supernatants collected from cells secreting the PirA^vp^/PirB^vp^ antibodies were collected and concentrated, and used to passively immunize shrimp to neutralize the toxins PirA^vp^ or PirB^vp^ associated with AHPND. Briefly, 10 μg of PirA^vp^ and PirB^vp^ antibodies, 7C12 and 9G10, respectively, were mixed with the shrimp feed, and fed to shrimp for three days consecutive days prior to experimentally infecting the shrimp with *V. parahaemolyticus* (containing toxins A and B), and resulting mortalities recorded for six days. Results showed significantly higher level of survival in shrimp fed with the PirB^vp^-9G10 antibody (60%) compared to the group fed the PirA^vp^-7C12 antibody (3%) and the control group (0%). This suggests that VLRB antibodies may be a suitable alternative to immunoglobulin-based antibodies, as passive immunization treatments for effective management of AHPND outbreaks within shrimp farms.

## 1. Introduction

Shrimp is an important aquatic food resource for human consumption worldwide, and are widely cultured to meet the growing demand for shrimp by consumers. This crustacean, as with other invertebrates, lacks adaptive immunity [1]. This is an important issue, especially since they are prone to acquiring infections and developing diseases when reared in aquaculture systems. Acute hepatopancreatic necrosis disease (AHPND), formerly known as early mortality syndrome, was first recognized as an emerging disease in China in 2009, and since being identified has spread to neighboring countries in Southeast Asia, including Vietnam in 2010, Malaysia in 2011, and Thailand in 2012. The disease has now reached as far as Mexico in early 2013 [2,3], the Philippines in 2015 [4] and South America in 2016 [5]. Three shrimp species appear susceptible to this disease, namely whiteleg shrimp (*Litopenaeaus/Penaeus vannamei*), black tiger shrimp (*Penaeus monodon*), and Chinese white shrimp (*Penaeus chinensis*) [6]. Affected shrimp have an empty gut and an atrophied pale hepatopancreas, which can be reduced in size by more than 50%. AHPND can cause up to 100% mortality within 20–30 days after the pond has been stocked with post-larvae shrimp [7]. The disease has resulted in huge economical losses for shrimp farmers globally. In Thailand alone, shrimp farmers experienced financial losses of $11.58 billion between 2010–2016, and total shrimp production fell by 54% between 2009 and 2014 due to AHPDH [8].

A unique strain of *Vibrio parahaemlyticus* is responsible for causing AHPND. *Vibrio parahaemlyticus* is a Gram-negative, halophilic bacterium found ubiquitously in warm marine and estuarine environments around the world [9,10]. The strains responsible for causing AHPND possess a 63 to 70 kDa plasmid that encodes binary toxins PirA^vp^/PirB^vp^, which are actually homologs of the *Photorhabdovirus* insect-related (Pir) toxins PirAB. These two toxins are secreted by the bacterium, and have been associated with the pathogenesis of the disease; they are considered to be the primary virulence factors involved in causing AHPND [11,12].

The variable lymphocyte receptor (VLR), composed of leucine-rich repeats (LRRSs), is a mediator of the humoral immune response in lamprey and hagfish [13]. As the VLRs mature, a repertoire of antigen-binding receptors are produced through the somatic diversification of the LRRs [14,15]. These antigen-binding receptors have three distinct types, namely VLRA, VLRB, and VLRC, which have been observed in both lamprey and hagfish. The VLRB has similarities to the B-cell receptor (BCR) in mammals. It is expressed on the cell membrane and is then secreted into the serum acting as a humoral agglutinin, making it the main component of the humoral immune response of jawless vertebrates with regards to antigen recognition [13,15,16]. Previous reports have shown that circulating antigen-specific VLRBs can be produced in response to an antigen (e.g., bacteriophages, *Brucella abortus*, human red blood cells and *Bacillus antracis* exosporium), with the VLRBs demonstrating both agglutinating and neutralizing activities [17,18].

A variety of methods have been investigated for controlling AHPND, including passive immunization. Here, we report on a VLRB antibody that we developed, which specifically recognizes and neutralizes the binary toxins produced by *V. parahaemolyticus* that are responsible for inducing the pathogenesis associated with AHPND in shrimp.

## 2. Materials and Methods

### 2.1. Construction of Toxin Plasmids

*Vibrio parahaemolyticus* (D2 strain) cells were cultured in brain heart infusion (BHI) broth containing 3% NaCl at 30 °C for 16 h. DNA was extracted from this bacterial cell culture using a G-spin™ Total DNA extraction kit (iNtRon Biotechnology, Seong-Nam, Korea). PirA^vp^ and PirB^vp^ were amplified using respective primers shown in Table 1. Specifically, PirA^vp^ was amplified with a 6× His tag added onto the C-terminal region of the sequence, then the amplified PirA^vp^ was flanked with Nde I and Sac I and cloned into pet32a vector (Novagen, Merck KGaA, Darmstadt, Germany). The PirB^vp^, on the other hand, was amplified to include three Strep tags on its C-terminal region and was cloned into a pet28b vector (Novagen, Merck KGaA, Darmstadt, Germany) between the Nco I and Xba I region. The Polymerase Chain Reaction (PCR) conditions used were as follows: initial denaturation: 95 °C, 5 min; denaturation: 95 °C, 20 s; annealing: 60 °C, 10 s; extension: 72 °C, 30 s; final extension: 72 °C, 5 min, for 30 cycles. The plasmids (pet32a-PirA^vp^ and pet28b-PirB^vp^) were transformed using BL21 competent cells. To check the veracity of the cloned PirA^vp^/PirB^vp^ plasmids, each plasmid was sequenced (Solgent, Korea) and the sequences aligned with the original PirA^vp^/PirB^vp^ sequence.

### 2.2. Expression and Purification of Recombinant Toxins

To induce the expression of PirA^vp^/PirB^vp^ proteins, BL21 cells harboring the pet32a-PirA^vp^ or pet28b-PirB^vp^ plasmids were grown overnight in Luria-Bertani (LB) broth with ampicillin (LB amp) and kanamycin (LB kan), respectively. The respective bacterial cultures were placed in fresh LB amp or LB kan, and grown to an OD 500 nm of 0.3, before adding 0.1 mM isopropyl thiogalactoside (IPTG) to the cultures for 4 h at 37 °C. The cells were collected by centrifugation at 3000× *g* for 5 min and resuspended in 1× phosphate-buffered saline (PBS) (with 200 mM NaCl) by vortexing. The cells were then subjected to three cycles of freeze-thawing to break the bacterial cell wall, sonicated with a 5 s pulse for 3 min on ice, and the soluble fractions collected by centrifuging the lysates at 3000× *g* for 10 min at 4 °C.

The soluble fractions were purified using affinity chromatography columns. The PirA^vp^ fractions were added to propylene column containing Ni-NTA agarose (Qiagen, Hilden, Germany), while the PirB^vp^ fractions were added to a column containing Streptactin resin (Iba Lifesciences^TM^, Gottingen, Germany). To remove any non-specific proteins bound to the resin, the columns were first washed with 1× PBS (with 10 mM imidazole), then the specific protein (PirA^vp^/PirB^vp^) was eluted from the column using elution buffer, 1× PBS (with 500 mM imidazole). To verify the correct sizes of the purified PirA^vp^/PirB^vp^ proteins, the eluted proteins were subjected to SDS-PAGE under reducing condition (samples (1:20 dilution) were treated with β–mercaptoethanol, boiled at 100 °C for 5 min, and run at 80 V for 20 min, 120 V for 90 min), after which the gels were stained with Coomassie blue.

The purified recombinant toxins were also subjected to Western blotting to further check their specificity. Briefly, the collected proteins were separated on 12% (PirA^vp^) and 8% (PirB^vp^) SDS-PAGE gel under reducing condition, then the separated proteins were transferred onto methanol-activated polyvinylidene fluoride (PVDF) membranes at 50 mA for 90 min. Blocking of the membrane was performed using 5% skimmed milk in 1× PBS mixed with 0.1% Tween 20. The membrane bearing the PirA^vp^ protein was incubated with (1:4 dilution) anti-6× His antibody produced by the lab, followed by (1:3000 dilution) HRP-conjugated goat anti-mouse IgG (Thermo Fisher Scientific, Waltham, MA, USA), while the membrane with the PirB^vp^ protein was incubated with (1:4000 dilution) streptactin-HRP antibody (Iba Lifesciences^TM^, Gottingen, Germany). The expression of the recombinant toxins was visualized using SuperSignal West Pico Chemiluminescent Substrate kit (Thermo Fisher Scientific). Large scale preparations of the respective proteins were performed once the correct sizes were verified. The proteins were dialyzed against 1× PBS to remove resin impurities and were quantified using Pierce^TM^ BCA protein Assay kit (Thermo Scientific^TM^) following the protocol provided in the kit. Finally, the quantified proteins were stored at −70 °C. These proteins were used as antigens in subsequent experiments.

### 2.3. Screening of VLRB Library

The cell line bearing the VLRB cDNA library [20] was seeded into twenty 96-well plates with 200 cells/well and grown to 100% cell confluency. The supernatants containing the recombinant VLRBs was collected and screened by ELISA, for which 96-well plates (Corning, Sigma-Aldrich, MO, USA) were coated with 200ng/well of PirA^vp^ or PirB^vp^ overnight at 4 °C. Unbound antigen was removed with 1× Tris buffered saline with Tween 20 (TBST: 10 mM Tris-HCl, 150 mM NaCl, 0.5% Tween 20, pH 8.0), and wells blocked with blocking buffer (5% skimmed milk in 1× TBST) for 1 h at room temperature, after which the plates were washed three times with 1× TBST. The collected recombinant VLRB supernatants were added to the plates for 1 h at room temperature. Binding of the recombinant VLRBs to their respective antigen was detected using mouse anti-VLRB IgG1 (11G5, produced in the lab) diluted (1:5) in blocking buffer, followed by a horseradish peroxidase (HRP)-conjugated goat anti-mouse IgG (Thermo Scientific, USA) (1:3000 dilution). After washing the plates again, 100 µL/well of substrate buffer containing 42 mM 3, 3′, 5, 5′-tetramethylbenzidine and 1% H_2_O_2_ was added for 20 min at room temperature, the reaction was stopped by adding 50 µL of stop solution (1 mM H_2_SO_4_) and the plate was then read at 450 nm using a microtiter plate reader. The ELISA was performed three times to ensure the specificity of the PirA^vp^ or PirB^vp^-specific VLRBs, with PirA^vp^-7C12 and PirB^vp^-9G10 showing the highest levels of specificity and were subsequently used in for further experiments.

### 2.4. Establishment of Cell Line Secreting Anti-PirA^vp^/PirB^vp^ Recombinant VLRBs

The cells secreting PirA^vp^/PirB^vp^-specific recombinant VLRBs, PirA^vp^-7C12, and PirB^vp^-9G10 were collected, then their respective DNA extracted using a DNA extraction kit (iNtRon Biotechnology) following the protocol provided with the kit. Amplification was performed using primer set LRRNT Sfi I/Stalk Sfi I (Table 1). The following PCR conditions used were initial denaturation: 95 °C, 5 min; denaturation: 95 °C, 20 s; annealing: 60 °C, 10 s; extension: 72 °C, 45 s; final extension: 72 °C, 5 min, for 30 cycles. The amplicons were purified using a DNA purification kit (iNtRon Biotechnology), digested with Sfi I enzymes for 1 h at 37 °C, and then were finally ligated into the Sfi I sites of the plasmid Δ514/VLR/kepta vector (developed in our lab). Colony PCR was performed to check the proper ligation of the DNA into the vector. The constructed plasmids, Δ514/VLR/kepta-PirA^vp^-7C12 and Δ514/VLR/kepta-PirB^vp^-9G10 were then transfected into human embryonic kidney (HEK) 293F cells in a 24-well plate using Lipofectamine 2000 (Invitrogen Life Technologies). After 4 h, the DNA-lipofectamine complexes were replaced with Dulbecco’s Modified Eagle Medium (DMEM) containing 2% fetal bovine serum (FBS). Two days after transfection, the supernatants were collected to further check the specificity of the VLRs by ELISA (as described above). Once specificity was established, large scale preparation of the supernatants was performed. These supernatants were designated as PirA^vp^-7C12 and PirB^vp^-9G10 antibody from here on. The VLRB antibodies were purified by affinity chromatography column using Streptactin resin (Iba Lifesciences^TM^, Gottingen, Germany), and then were freeze-dried using a refrigerated vacuum. All the freeze-dried VLRB antibodies were stored in −70 °C until used.

### 2.5. Bacterial Challenge Test

*Litopenaeus vannamei* post-larvae (*n* = 100, 0.1 ± 0.03 g) were transferred into three 250-L tanks corresponding to the two experimental groups (PirA^vp^-7C12 and PirB^vp^-9G10 antibody) and one control group fed no antibody (negative control). The shrimp were fed with antibody added to the shrimp diet (10 µg of respective antibody per gram of feed (equivalent to 8% bodyweight/day)) every day for three days prior to the challenge test. After three days, the shrimp were challenged by immersion with an AHNPD-causing strain of *V. parahaemolyticus.* To be more specific, each treatment group containing 30 shrimp, were immersed in 1.5 water (25 ppm salinity) containing 10^7^ colony forming units (cfu) mL^−1^ of *V. parahaemolyticus* for 15 min. Determination of colony forming unit (CFU) for *V. parahaemolyticus* was remunerated with a standard plate count technique using thiosulfate-citrate-bile salts-sucrose agar. The challenged shrimp and bacterial solution were poured into a new aquarium containing 100 volume of clean water. The final volume was 15 L, which contained 10^5^ cfu/mL of *V. parahaemolyticus*. Shrimp were maintained under these conditions for another 24 h. After 24 h, 100% water was replaced with fresh clean water (25 ppm salinity). The shrimp (*n* = 30 per group) were then transferred into new15 L tanks, where they were monitored for six days. They were continuously fed with the respective experimental feed diet containing the antibody during this time. Mortality data were obtained from two separate trials.

### 2.6. Statistical Analysis

Survival data were statistically analyzed via Kaplan-Meier with the Chi-square test using GraphPad Prism v.5 software. Differences between groups were considered significant when ** *p* < 0.001.

## 3. Results

### 3.1. Expression of Recombinant Toxins

**Figure 1:**
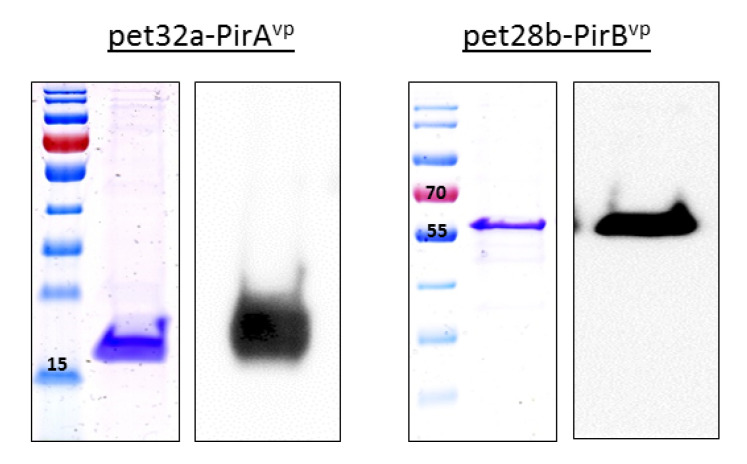
The expression of PirA^vp^ and PirB^vp^ was verified through Coomassie staining and Western Blotting. After purification using His resin, **PirA^vp^** was evident as a band at ~15 kDa (using His Ab for detection), while PirB^vp^ was purified using Streptactin resin and a band evident at ~55 kDa size (using Streptactin-HRP for Western blot detection).

Successful transformation of toxin plasmids produced a band corresponding to 336 bp for pet32a-PirA^vp^ and 1317 bp for pet28b-PirB^vp^ (Supplementary Figure S1), while the results of the Western blot and Coomassie blue staining showed distinct bands at appropriately 15 and 55 kDa for PirA^vp^ and PirB^vp^, respectively (Figure 1).

### 3.2. Specificity of Established Toxin-Specific VLRBs

**Figure 2:**
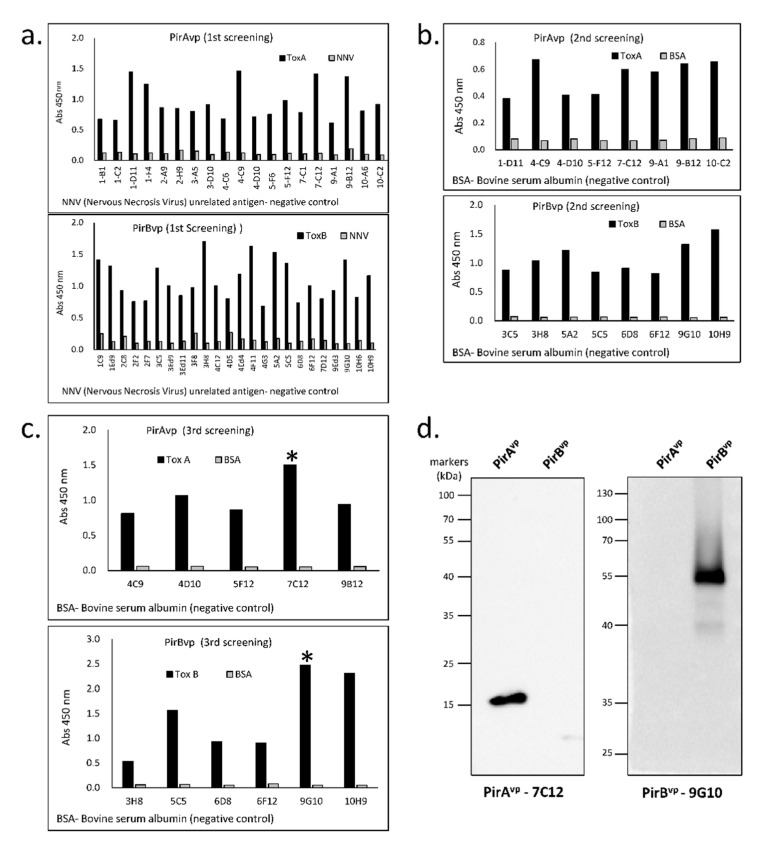
ELISA screening and Western blotting of recombinant VLRBs to check specificity to PirA^vp^ and PirB^vp^. (**a**) first screening, (**b**) second screening, (**c**) third screening, and (**d**) blotting results show specificity of the VLRBs, PirA^vp^-7C12, and PirB^vp^-9G10, with band size 15 kDa and 55 kDa, corresponding to PirA^vp^ and PirB^vp^, respectively. The * corresponds to the respective VLRB-specific PirA^vp^ and PirB^vp^ used in succeeding experiments.

The capacity of the toxin-specific VLRBs to recognize their respective PirA^vp^ and PirB^vp^ antigens was verified by ELISA and Western blotting. Of the twenty 96-well plates that that were screened in the first round of ELISA screening, only 19 wells showed high binding with PirA^vp^ and 24 wells with PirB^vp^ (Figure 2a). Positive wells were serially diluted to obtain single cells, which were again screened by ELISA. In the second screening, eight cells from each of the PirA^vp^ and PirB^vp^ groups displayed a binding affinity to the antigen (Figure 2b). After the third round of screening, only one antibody for each group with the highest binding capacity was selected, namely PirA^vp^-7C12 and PirB^vp^-9G10 (Figure 2c). Western blot analysis of these two selected toxin-specific VLRBs showed specific binding to the respective toxin antigens, thereby, verifying the specificity of these antibodies for recognizing either PirA^vp^ or PirB^vp^, respectively (Figure 2d).

### 3.3. Protective Efficiency of Toxin-Specific VLRBs in Shrimp Infected with V. parahaemolyticus

**Figure 3:**
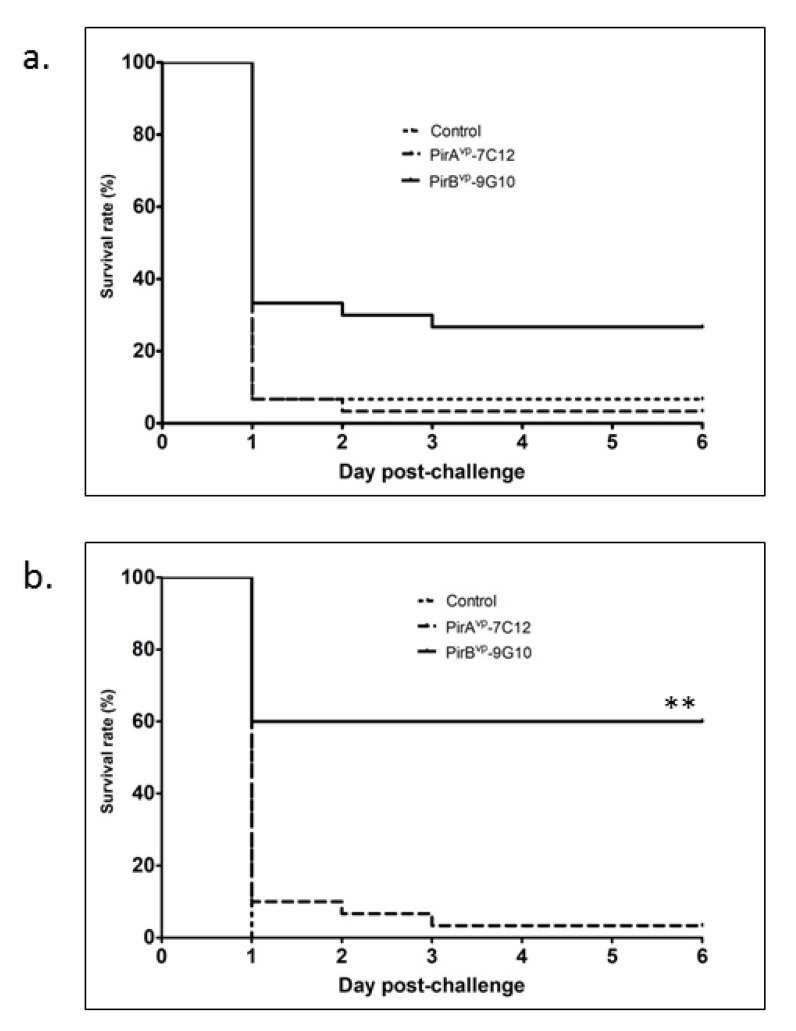
Survival rate of shrimp after bacterial challenge. Shrimp fed with or without VLRB antibodies were challenged with *Vibrio parahaemolyticus* by immersion and mortality was recorded for six days. (**a**) first trial and (**b**) second trial. ** Statistically significant *p* < 0.001.

Based on the results collected, the level of survival in the first experimental trial in which shrimp were fed with PirB^vp^-9G10 antibody was 26.7%, which was significantly higher than the group fed with PirA^vp^-7C12 antibody (3%) and the negative control (6%) (Figure 3a). In the replicate trial, results were even more pronounced, wherein the group fed with PirB^vp^-9G10 antibody demonstrated a 60% survival, in contrast to the group fed PirA^vp^-7C12 or the negative control group that exhibited 3% and 0% survival, respectively (Figure 3b). This particular dataset is statistically significant at ** *p*< 0.001, indicating the protective effect of the PirB^vp^-9G10 antibody.

## 4. Discussions

The demand for farmed shrimp continues to grow faster than any other aquaculture species in the global setting, with most of the shrimp being produced coming from Asia. In the latest statistics presented by the Food and Agriculture Organization of the United Nations (FAO), it was estimated that the global production of farmed shrimp is growing at an annual rate of 6% [21]. With this growing trend, one of the ways to address the increasing demand for shrimp is to use high density stocking within shrimp farms. As a result of this intensification, farmed shrimp are continually being plagued by disease, which has greatly affected shrimp production. Thus, the development of safe and efficacious treatment for diseases such as AHPND has been the subject of increased research in recent years. The use of antibiotics to treat bacterial infections in industrial aquaculture has been opposed despite their effectiveness, due to antibiotic usage giving rise to antibiotic-resistant strains of bacteria. Vaccination is currently considered to be the most effective strategy for controlling infections in aquaculture; however, shrimp do not possess acquired immunity, necessary to induce a memory response to the vaccine. Also, they are too fragile to be vaccinated by intraperitoneal injection, the traditional route of vaccine delivery, which is both labor-intensive and stressful, so oral administration of antibodies to passively immunize shrimp is an attractive alternative.

The potential of using passive immunization to protect shrimp against infections has been explored previously, with varying degrees of success. Previous studies have used chicken antibodies (immunoglobulin Y–IgY) to develop passive vaccination protocols for shrimp; chickens are able to efficiently produce large amounts of IgY in their eggs [22]. Gao et al. (2016) showed egg yolk powder containing antibodies against *V. harveyi* and *V. parahaemolyticus* orally administered to white shrimp, *Litopenaeus vannamei* to be effective for reducing subsequent *Vibrio* infections. More specifically, the antibodies showed an inhibiting effect on both bacteria in vitro, and in vivo. When the anti-*Vibrio* antibodies, present in egg yolk were encapsulated in β-cyclodextrin, and fed to shrimp, lower mortality was observed in zoeae, mysis and post-larva, and lower bacterial load recorded in post-larva compared to shrimp fed with normal egg powder (i.e., from non-immunized chickens) [23]. Likewise, in another study Indian white shrimp were fed with an anti-*V. harveyi* IgY, obtained from eggs of chicken immunized with *V. harveyi,* for 30 and 60 days and subsequently challenged with a virulent strain of *V. harveyi* and their hematological and immunological parameters evaluated. The group fed with the edible IgY exhibited a significant increase in total hemocyte counts (THC), and increased levels of coagulase, oxyhemocyanin, prophenoloxidase, intracellular superoxide anion production, lysozyme activity, phagocytosis, and bacterial agglutinin. Furthermore, the group fed with the anti-*V. harveyi* IgY-coated feeds had a lowered *Vibrio* load compared to control shrimp and had an improved immune response against the *V. harveyi* challenge [24]. In another study of note, outer membrane proteins (OMPs) obtained from *V. parahaemolyticus* were used to immunize hens to produce *V. parahaemolyticus* OMP IgY antibodies. These were then incorporated into extruded pellet diets and fed to white pacific shrimp, experimentally infected with the bacterium. Lower bacterial loads were measured in the muscle of shrimp fed with the specific IgY incorporated into their diets, compared with the control group fed with non-specific IgY from non-immunized chickens. Along with those results, the superoxide dismutase (SOD) activity in the muscle of the IgY-fed shrimp was significantly higher than the control group [25]. In line with these studies, a potential way to treat *Vibrio* sp. causing AHPND is to target the toxins it secretes while infecting its host. In one such study, IgY from eggs of hens immunized with recombinant PirA- and PirB-like toxins (the virulent toxins released by AHPND-causing *V. parahaemolyticus*) was administered orally to shrimp by feeding them egg yolk powder containing the IgY; 87% survival was noted in shrimp fed with the anti-PirA-IgY-coated feed after challenging the shrimp with the bacterium [26].

The results of the studies described above, indicate the potential of using an edible antibody as a means of passively immunizing shrimp to help them fight infection. In our current study, we developed VLRB antibodies that specifically recognized PirA^vp^ and PirB^vp^, which could potentially “neutralize” the effect of these virulence toxins. Although the exact mechanism of action of these toxins is still unclear, the presence of the plasmid (pVA1) encoding these toxins in all AHPND-causing strains of *V. parahaemolyticus* indicates that they are causative factors involved in the disease process. The PirA^vp^/PirB^vp^ toxins are known to be homologs of the insecticidal *Photorhabdus* insect-related (Pir) binary toxin PirAB that exhibits pore-forming activity in insects, thus suggesting that PirA^vp^ and PirB^vp^ might function similarly. In a previous report, both Pir A and Pir B are needed to be present in insect larvae to induce mortality [27]. Since these binary toxins have been discovered together in *V. parahaemolyticus*, it would seem reasonable that they are also both essential for the onset of symptoms associated with AHPND.

Structural analysis of the PirA^vp^/PirB^vp^ binary toxins suggested strong similarity with *Bacillus thuringiensis* Cry toxins [11,28]. The N-terminal domain of PirB^vp^, as with the Cry domain I, contains a bundle of α-helices and in the center of this α-bundle, there are abundant hydrophobic residues. Specifically, the hydrophobic α-helix 8 is lodged within a bunch of amphipathic helices; this “inside-out membrane fold” is a typical characteristic of other pore-forming toxins, which can switch between soluble and transmembrane conformations [29]. Such characteristics strongly implicate that the N-terminal region of PirB^vp^ has the capability to form a pore in the cell membrane that leads to cell death [11]. On the other hand, the C-terminal of PirB^vp^, similar to the Cry domain II, has three antiparallel β-sheets and contains an immunoglobulin-like folding domain that plays a role in protein-protein or protein-ligand interactions [30], thereby indicating that the PirB^vp^ C-terminal plays a similar functional role. Moreover, since this domain can interact with insect receptors in its insect homolog, the similarity in structure suggests that the PirB^vp^ C-terminal is also a receptor binding domain [31,32,33,34,35,36,37]. Understanding the structural conformation of PirB^vp^ and its role in the pathogenicity of AHPND is very important, as this could help scientists in formulating potential treatments for the disease.

Several notable studies had demonstrated the potential role of VLRBs in neutralizing certain viruses such as avian influenza virus H9N2 [38], viral hemorrhagic septicemia virus [19] and nervous necrosis virus [20], and results are compelling enough to promote their usage as a therapeutic agent against bacterial and viral infections. In the current study, the high level of survival in the group fed with the PirB^vp^-9G10 antibody after the AHPND-challenge, suggests that the VLRB antibody can provide protection against a *V. parahaemolyticus* infection in shrimp. The difference in the survival rate between the two in vivo feeding trials performed in the current study, might reflect a difference in the amount of feed consumption by the shrimp within the two feeding trials [39,40]. Our finding appears to be in contrast with the results of a previous study, wherein shrimp fed with diet containing anti-PirA-IgY demonstrated consistently higher levels of survival compared with anti-PirB-IgY-fed and control groups [25]. We speculate that the reason behind the effectiveness of the PirB^vp^-9G10 antibody in our study might be due to the abundant hydrophobic residues in the structure of PirB^vp^ that our VLRB antibody readily recognizes and which the PirA^vp^ noticeably lacks. VLRBs are known to have high binding capacity with hydrophobic structures such as carbohydrates and glycoproteins that form hydrophobic clusters [41], thus contributing to the efficiency of PirB^vp^-9G10 antibody reaction with the PirB^vp^ toxin. Although the use of the PirA^vp^-7C12 antibody may not be as effective as the PirB^vp^-9G10 antibody, we still consider its potency as a therapeutic agent, and we plan to do further studies focusing on its use. However, in general, our results clearly suggest that the PirB^vp^-9G10 VLRB antibody can improve shrimp survival against *V. parahaemolyticus* by simply targeting the virulent toxin PirB^vp^.

In summary, the results of a previous report showing that only the PirB^vp^ toxin could induce histological signs of AHPDH [11], and another stating that the virulence of AHPND relies heavily on the amount of toxins secreted by the bacterial cells [42], greatly substantiates the aim of our study to develop new therapeutic agents for AHPND targeting the PirB^vp^ toxin. Furthermore, the efficacy of VLRB antibodies as immunogenic agents to passively immunize shrimp reared at high stocking densities could significantly help the shrimp industry to combat outbreaks of AHPND.

## Figures and Tables

**Table 1:** Primer Sequences.

Name	Sequence (5′-3′)	Reference/Accession No.
For PirAB^vp^ amplification		
p32-ToxA/Nco1-Fwd	TATACCATGGAGTAACAATATAAAACATG	AB972427.1
p32-ToxA/Sac1-Rev	GATGAGCTCTTAGTGGTAATAGATTGTAC	AB972427.1
p2b-ToxB/Nco1-Fwd:	ATACCATGGGCATGACTAACGAATACGTTGTAACAATGTC	AB972427.1
p2b-ToxB-TStrep/**STOP**/Sac1/Rev	AGCGAGCTCTCACTTTTCAAACTGCGGATGGCTCCACGCGCTGCCACCGCTACCGCCACCGCTACCGCCACCTTTCTCAAACTGTGGATGGCT	AB972427.1
For cell line		
LRRNT Sfi I forward	AGGCCACCGGGGCCTGTCCTTCACGGTGTTCCTG	[19]
Stalk Sfi I reverse	TGGCCCAGAGGCCCGCGTTCATGACACGGCCGA	[19]

## Data Availability

The data presented in this study are available on request from the corresponding author. The data are not publicly available due to privacy reasons.

## Supplementary Materials

The following are available online at https://www.mdpi.com/2076-393X/9/1/55/s1, Figure S1: Colony PCR to check proper cloning of pet32a-PirA^vp^ and pet28b-PirB^vp^. Checking presence of inserts by amplifying the cloning site using gene-specific primers. Band size 336 bp and 1317 bp were observed, which correspond to PirA^vp^ and PirB^vp^, respectively. One clone from each gene was sent for sequencing to further check the identity of the gene inserted in the plasmid.

## References

[1] Zhang Y., Qiu L.M., Song L.S., Zhang H., Zhao J.M., Wang L.L., Yu Y.D., Li C.H., Li F.M., Xing K.Z. (2009). Cloning and characterization of a novel C-type lectin gene from shrimp *Litopenaeus vannamei*. Fish Shellfish Immunol..

[2] Gomez-Gil B., Soto-Rodriguez S., Lozano R., Betancourt-Lozano M. (2014). Draft genome sequence of *Vibrio parahaemolyticus* strain M0605, which causes severe mortalities of shrimps in Mexico. Genome Announc..

[3] Nunan L., Lightner D., Pantoja C., Gomez-Jimenez S. (2014). Detection of acute hepatopancreatic necrosis disease (AHPND) in Mexico. Dis. Aquat. Org..

[4] De la Peña L.D., Cabillon N.A., Catedral D.D., Amar E.C., Usero R.C., Monotilla W.D., Calpe A.T., Fernandez D.D., Saloma C.P. (2015). Acute hepatopancreatic necrosis disease (AHPND) outbreaks in *Penaeus vannamei* and *P. monodon* cultured in the Philippines. Dis. Aquat. Org..

[5] Restrepo L., Bayot B., Betancourt I., Pinzon A. (2016). Draft genome sequence of pathogenic bacteria *Vibrio parahaemolyticus* strain Ba94C2, associated with acute hepatopancreatic necrosis disease isolate from South America. Genom. Data.

[6] NACA (2012). Report of the Asia Pacific Emergency Regional Consultation on the Emerging Shrimp Disease: Early Mortality Syndrome (EMS)/Acute Hepatopancreatic Necrosis Syndrome (AHPNS).

[7] De Schryver P., Defoirdt T., Sorgeloos P. (2014). Early mortality syndrome outbreaks: A microbial management issue in shrimp farming. PLoS Pathog..

[8] Lai H.C., Ng T.H., Ando M., Lee C.T., Chen I.T., Chuang J.C., Mavichak R., Chang S.H., Yeh M.D., Chiang Y.A. (2015). Pathogenesis of acute hepatopancreatic necrosis disease (AHPND) in shrimp. Fish Shellfish Immunol..

[9] Broberg C.A., Calder T.J., Orth K. (2011). *Vibrio parahaemolyticus* cell biology and pathogenicity determinants. Microbes Infect..

[10] Zhang L., Orth K. (2013). Virulence determinants for *Vibrio parahaemolyticus* infection. Curr. Opin. Microbiol..

[11] Lee C.T., Chen I.T., Yang Y.T., Ko T.P., Huang Y.T., Huang J.Y., Huang M.F., Lin S.J., Chen C.Y., Lin S.S. (2015). The opportunistic marine pathogen *Vibrio parahaemolyticus* becomes virulent by acquiring a plasmid that expresses a deadly toxin. Proc. Natl. Acad. Sci. USA.

[12] Sirikharin R., Taengchaiyaphum S., Sanguanrut P., Chi T.D., Mavichak R., Proespraiwong P., Nuangsaeng B., Thitamadee S., Flegel T.W., Sritunyalucksana K. (2015). Characterization and PCR detection of binary, Pir-like toxins from *Vibrio parahaemolyticus* isolates that cause acute hepatopancreatic necrosis disease (AHPND) in shrimp. PLoS ONE.

[13] Poole J.R.M., Paganini J., Pontarotti P. (2017). Convergent evolution of the adaptive immune response in jawed vertebrates and cyclostomes: An evolutionary biology approach based study. Dev. Comp. Immunol..

[14] Alder M.N., Rogozin I.B., Iyer L.M., Glazko G.V., Cooper M.D., Pancer Z. (2005). Diversity and function of adaptive immune receptors in a jawless vertebrate. Science.

[15] Herrin B.R., Alder M.N., Roux K.H., Sina C., Ehrhardt G.R.A., Boydston J.A., Turnbough C.L., Cooper M.D. (2008). Structure and specificity of lamprey monoclonal antibodies. Proc. Natl. Acad. Sci. USA.

[16] Li J., Das S., Herrin B.R., Hirano M., Cooper M.D. (2013). Definition of a third VLR gene in hagfish. Proc. Natl. Acad. Sci. USA.

[17] Fujii T., Nakagawa H., Murakawa S. (1979). Immunity in lamprey. I. Production of haemolytic and haemagglutinating antibody to sheep red blood cells in Japanese lampreys. Dev. Comp. Immunol..

[18] Alder M.N., Herrin B.R., Sadlonova A., Stockard C.R., Grizzle W.E., Gartland L.A., Gartland G.L., Boydston J.A., Turnbough C.L., Cooper M.D. (2008). Antibody responses of variable lymphocyte receptors in the lamprey. Nat. Immunol..

[19] Lee J.S., Kim J., Im S.P., Kim S.W., Lazarte J.M., Jung J.W., Gong T.W., Kim Y.R., Lee J.H., Kim H.J. (2018). Generation and characterization of hagfish variable lymphocyte receptor B against glycoprotein of viral hemorrhagic septicemia virus (VHSV). Mol. Immunol..

[20] Jung J.W., Lee J.S., Kim J., Im S.P., Kim S.W., Lazarte J., Kim Y.R., Chun J.H., Ha M.W., Kim H.S. (2020). Characterization of Hagfish (*Eptatretus burgeri*) Variable Lymphocyte Receptor-Based Antibody and Its Potential Role in the Neutralization of Nervous Necrosis Virus. J. Immunol..

[21] www.fao.org/in-action/globefish/market-reports/resource-detail/en/c/1261310.

[22] Gottstein B., Hemmeler E. (1985). Egg yolk immunoglobulin Y as an alternative antibody in the serology of echinococcosis. Z. Parasitenkd..

[23] Gao X., Zhang X., Lin L., Yao D., Sun J., Du X., Zhang Y. (2016). Passive Immune-Protection of *Litopenaeus vannamei* against *Vibrio harveyi* and *Vibrio parahaemolyticus* Infections with Anti-Vibrio Egg Yolk (IgY)-Encapsulated Feed. Int. J. Mol. Sci..

[24] Kumaran T., Thirumalaikumar E., Lelin C., Palanikumar P., Michaelbabu M., Citarasu T. (2018). Physicochemical properties of anti *Vibrio harveyi* egg yolk antibody (IgY) and its immunological influence in Indian white shrimp *Fenneropenaeus indicus*. Fish Shellfish Immunol..

[25] Hu B., Yang X., Guo E., Zhou P., Xu D., Qi Z., Deng L.J. (2019). The preparation and antibacterial effect of egg yolk immunoglobulin (IgY) against the outer membrane proteins of *Vibrio parahaemolyticus*. Sci. Food Agric..

[26] Nakamura R., Pedrosa-Gerasmio I.R., Alenton R.R.R., Nozaki R., Kondo H., Hirono I. (2019). Anti-PirA-like toxin immunoglobulin (IgY) in feeds passively immunizes shrimp against acute hepatopancreatic necrosis disease. J. Fish Dis..

[27] Waterfield N., Kamita S.G., Hammock B.D., Ffrench-Constant R. (2005). The Photorhabdus Pir toxins are similar to a developmentally regulated insect protein but show no juvenile hormone esterase activity. FEMS Microbiol. Lett..

[28] Lin S.J., Hsu K.C., Wang H.C. (2017). Structural Insights into the Cytotoxic Mechanism of *Vibrio parahaemolyticus* PirA^vp^ and PirB^vp^ Toxins. Mar. Drugs..

[29] Peraro M.D., van der Goot F.G. (2015). Pore-forming toxins: Ancient, but never really out of fashion. Nat. Rev. Microbiol..

[30] Kitami M., Kadotani T., Nakanishi K., Atsumi S., Higurashi S., Ishizaka T., Watanabe A., Sato R. (2011). *Bacillus thuringiensis* Cry Toxins Bound Specifically to Various Proteins via Domain III, Which Had a Galactose-Binding Domain-Like Fold. Biosci. Biotechnol. Biochem..

[31] Bravo A., Gill S.S., Soberón M. (2007). Mode of action of *Bacillus thuringiensis* Cry and Cyt toxins and their potential for insect control. Toxicon.

[32] Pardo-López L., Soberón M., Bravo A. (2013). *Bacillus thuringiensis* insecticidal three-domain Cry toxins: Mode of action, insect resistance and consequences for crop protection. FEMS Microbiol. Rev..

[33] Adang M., Crickmore N., Jurat-Fuentes J. (2014). Diversity of *Bacillus thuringiensis* Crystal Toxins and Mechanism of Action. Adv. Insect Physiol..

[34] Bravo A., Gómez I., Porta H., García-Gómez B.I., Rodriguez-Almazan C., Pardo L., Soberón M. (2012). Evolution of *Bacillus thuringiensis* Cry toxins insecticidal activity. Microb. Biotechnol..

[35] Grochulski P., Masson L., Borisova S., Pusztai-Carey M., Schwartz J.L., Brousseau R., Cygler M. (1995). *Bacillus thuringiensis* CryIA(a) insecticidal toxin: Crystal structure and channel formation. J. Mol. Biol..

[36] Soberón M., Pardo L., Muñóz-Garay C., Sánchez J., Gómez I., Porta H., Bravo A. (2010). Pore formation by Cry toxins. Adv. Exp. Med. Biol..

[37] Pigott C.R., Ellar D.J. (2007). Role of receptors in *Bacillus thuringiensis* crystal toxin activity. Microbiol. Mol. Biol. Rev..

[38] Im S.P., Kim J., Lee J.S., Kim S.W., Jung J.W., Lazarte J.M.S., Kim J.Y., Kim Y.R., Lee J.H., Chong R.S.M. (2018). Potential use of genetically engineered variable lymphocyte receptor B specific to avian influenza virus H9N2. J. Immunol..

[39] Loc Tran L., Nunan L., Redman R., Mohney L., Pantoja C., Fitzsimmons K., Lightner D. (2013). Determination of the infectious nature of the agent of acute hepatopancreatic necrosis syndrome affecting penaeid shrimp. Dis. Aquat. Org..

[40] Joshi J., Srisala J., Truong V.T., Chen I.-T., Nuangsaeng N., Suthienkul O., Lo C.L., Flegel T.W., Sritunyalucksana K., Thitamadee S. (2014). Variation in *Vibrio parahaemolyticus* isolates from a single Thai shrimp farm experiencing an outbreak of acute hepatopancreatic necrosis disease (AHPND). Aquaculture.

[41] Kim J., Im S.P., Lee J.S., Lazarte J., Kim S.W., Jung J.W., Kim J.Y., Kim Y.R., Lee S., Kim G.J. (2018). Globular-shaped variable lymphocyte receptors B antibody multimerized by a hydrophobic clustering in hagfish. Sci. Rep..

[42] Tinwongger S., Nochiri Y., Thawonsuwan J., Nozaki R., Kondo H., Awasthi S., Hinenoya A., Yamasaki S., Hirono I. (2016). Virulence of acute hepatopancreatic necrosis disease PirAB-like relies on secreted proteins not on gene copy number. J. Appl. Microbiol..

