# Supplementary material for: Passive Immunization with Recombinant Antibody VLRB-PirAvp/PirBvp—Enriched Feeds against Vibrio parahaemolyticus Infection in Litopenaeus vannamei Shrimp
Source: Vaccines (Basel). 2021 Jan 16;9(1):55. doi: 10.3390/vaccines9010055 (PMC7829966; doi:10.3390/vaccines9010055)
Supplement: Supplementary file 1 [file vaccines-09-00055-s001.pdf]

Supplementary figure 1.

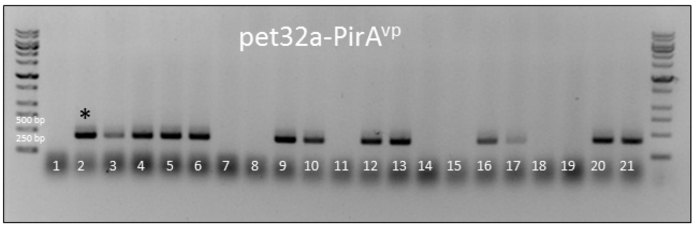

* Correct sequence.

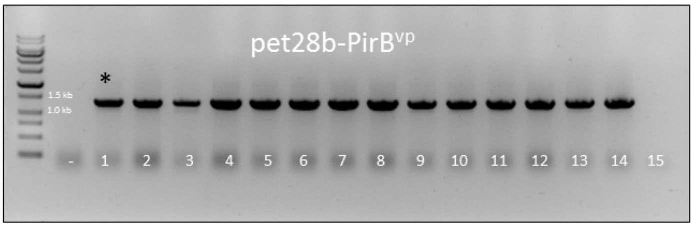

* Correct sequence.

Supplementary Figure 1. Colony PCR to check proper cloning of pet32a-PirA^{vp} and pet28b-PirB^{vp}. Checking presence of inserts by amplifying the cloning site using gene-specific primers. Band size 336 bp and 1317 bp were observed, which correspond to **PirA^{vp} and PirB^{vp},** respectively. One clone from each gene was sent for sequencing to further check the identity of the gene inserted in the plasmid.
